# Developmental framework for a desktop hydrogeomorphic wetland functional assessment derived from field-based data

**DOI:** 10.1007/s10661-024-12373-z

**Published:** 2024-01-30

**Authors:** Peter J. Backhaus, Denice H. Wardrop, Gregory W. McCarty, Robert P. Brooks

**Affiliations:** 1 Riparia, Department of Geography, Pennsylvania State University, 302 Walker Building, University Park, State College, PA 16802 USA; 2 Chesapeake Research Consortium, Riparia, Department of Geography, Pennsylvania State University, 302 Walker Building University Park, State College, PA 16802 USA; 3 Hydrology and Remote Sensing Laboratory, USDA Agricultural Research Service (ARS), 10300 Baltimore Avenue, Beltsville, MD 20705 USA

**Keywords:** Wetlands, Ecosystem services, Functional assessment, HGM, Remote sensing

## Abstract

With loss of wetlands and their associated ecosystem services within landscapes, it is imperative to be able to understand the change in ecological functions underlying these services. Field-based functional assessments can produce a range of specific scores among a robust set of functions but are time and cost prohibitive as the number of wetlands assessed increases. Remote-based functional assessments are an alternative for broad scale assessments, but trade-off cost for limitations in scoring and functional assemblage. To address these concerns, we created a framework for the development of the Hydrogeomorphic Remote Assessment of Wetland Function (HGM-RAWF). Rooted in the hydrogeomorphic approach of an existing field-based functional assessment and its underlying models, this remote functional assessment substitutes field-based assessment methods with remotely assessed proxies. As potential remote proxies were determined through literature review and statistically screened for use in the remote assessment, a field-based reference wetland database of 222 freshwater wetlands in the Mid-Atlantic Region provided a baseline by which remote data could be compared and calibrated. The resulting HGM-RAWF protocol remotely assesses seven hydrology and biogeochemistry functions in the Mid-Atlantic with assessment scores similar to its field-based counterparts. With noted limitations, the HGM-RAWF framework provides the means to create desktop functional assessments across broad geographic scales with the diversity and specificity of field-based assessments at the reduced costs associated with remote assessments. Its basis in the HGM approach and use of public spatial datasets allows the framework to be adopted regionally and can be used as a model for national wetland functional assessment.

## Introduction

Wetlands are among the most productive sources of ecosystem services per unit area, including a suite of services unique to this type of ecosystem (Costanza et al., 1997). These ecosystems provide per-unit area economic values greater than those of upland and waterbody ecosystems (Mitsch & Gosselink, 2015) through services such as the provisioning of food and fiber, climate regulation, flood desynchronization and storage, water purification, and biodiversity support (Millennium Ecosystem Assessment, 2005). Additionally, non-monetary socio-cultural benefits including recreational, educational, and spiritual services make wetlands an important asset to humanity and its well-being (Millennium Ecosystem Assessment, 2005).

Ecosystem services are derived from ecosystem functions, defined as the physical, biological, and chemical processes that maintain the structure of the ecosystem (Boyd & Wainger, 2002; Ringold et al., 2011). In wetlands, ecosystem services such as water storage/desynchronization, water quality improvement, and wildlife habitat stem from hydrologic, biogeochemical, and biodiversity functions, respectively. With wetlands acting as landscape “sinks” due to their geomorphic position, their functional capacity is vulnerable to degradation from negative influences in their contributing landscape (Brinson, 1993a; Mitsch & Gosselink, 2000). Such changes lead to a decrease in functional assemblage and/or performance and their associated ecosystem services, and the condition of the wetland is decreased as the loss of function compromises the structure of the ecosystem. Compounding this functional degradation is the outright loss of wetland ecosystem area due to actions such as draining or filling. In the contiguous USA, over 50% of wetland area has been converted to other land uses (Dahl, 1990), a trend that has slowed in recent decades but still contributes a net loss of area (Dahl, 2011). While wetland creation, restoration, enhancement, and other conservation practices are preserving or increasing wetland area, much of this area provides a less than optimal suite and/or level of ecosystem services and may exhibit degraded condition (Brooks et al., 2005; Gebo & Brooks, 2012; McLaughlin, & Cohen., M.J., 2013; Zedler & Kercher, 2005).

To understand changes in wetland function and their associated changes in ecosystem services, wetland functional assessments are used to provide scores by which wetland functional assemblage and performance can be compared. Field-based, site-specific functional assessments are likely the most implemented means of assessment currently, with hydrogeomorphic (HGM) methods remaining the dominant approach since their development by the US Army Corps of Engineers (ACOE) in the early 1990s (Brinson, 1993b; Smith et al., 1995). HGM functional assessments use models of the physical, chemical, and biological characteristics of a wetland to determine functional assessment scores. These scores are calibrated relative to reference standards, the highest level of sustained functional capacity exhibited by that HGM subclass, established through a set of reference wetland sites within each subclass that span the subclass’ range of functional capacity. Within this set of reference wetlands, a subset of these sites expressing the attributes that are consistent with the highest level of sustained functional capacity are denoted as reference standard wetlands and are used to establish the reference standards. Field-based HGM functional assessments typically require several hours of sampling by a small team at each individual site being assessed followed by an assessment protocol (e.g., Wardrop et al., 2004 in Brooks, 2004) that utilizes functional models for each function assessed. These assessments generally rely on GIS landscape assessment data (EPA Level 1 Landscape Assessment) and rapid field assessment methods (Level 2 Rapid Assessment) that are combined with intensive site sampling and assessment protocols (Level 3 Intensive Site Assessments) to produce an assessment score for each functional model present for that wetland subclass.

The upfront time and labor costs associated with the establishment of reference wetlands, in addition to the costs of implementing the sampling and assessment of wetlands once the assessment has been calibrated, make field-based functional assessment impractical as the number of wetlands and/or geographic area increases. While regional and national efforts have been made to track changes in wetland area, habitat, and condition (e.g., National Wetland Inventory [NWI] and National Wetland Condition Assessment [NWCA]), the ability to perform functional assessment of wetlands across watersheds, regions, and other broad geographic areas is currently limited. The NWI and NWCA are rooted in spatial data or statistical interpolation of field sampling, respectively, but no such regional or national methods for functional assessment have been developed. To create a baseline of existing wetland ecosystem services and their ecosystem function, as well as be able to observe change in these services and functional performance over time, we must have the ability to inventory wetland functions across larger geographic areas such as watersheds, states, regions, and nationally (Comer & Faber-Langendoen, 2013).

At these broad scales, functional assessments based on remote sensing data and statistical extrapolation are a more attractive option than traditional field-based methods. Several remote-based wetland functional assessments exist, but the use of remote data often limits the number of functions assessed, the specificity of results (e.g., limited categorical outputs), and the applicable regions for which the remote assessment can be applied. In a prior study by Backhaus et al. (2020), a comparison of a HGM functional assessment for Pennsylvania, USA, wetlands (Brooks, 2004) and a remote-based functional assessment used in the Mid-Atlantic Region (Watershed-based Preliminary Assessment of Wetland Function [W-PAWF; Tiner, 2003]) found that the remote assessment could detect only a limited range of functional assemblage and performance level in wetlands known to have more diverse and specific scores through the field-based assessment. However, potential overlap between the field-based and remote-based methods were found, demonstrating the potential creation of a remote sensing-based functional assessment based in the framework of field-based HGM functional assessment that would allow the limitations of existing remote functional assessments to be addressed. It was therefore theorized that the linking of data from reference wetlands, level 2 rapid assessments, and/or level 3 intensive assessments to remote sensing and other spatial data (i.e., using remote data as proxies for field sampling) could provide an avenue by which more accurate remote-based functional assessment could be developed.

As such, this study sought to build upon the findings of Backhaus et al. (2020) through the development of a Hydrogeomorphic Remote Assessment of Wetland Function (HGM-RAWF) protocol that utilizes remote sensing and spatial data as proxies for current field-based assessment methods, such that a suite of wetland functional assemblages and performances can be quantitatively inventoried at watershed and regional scales. This assessment system focuses on freshwater wetlands of the Mid-Atlantic Region, given availability of wetland data for several of its ecoregions and an existing, trusted field-based functional assessment framework for the region (Brooks, 2004). This field-based data also allowed for the validation of the remote-based functional models, allowing direct comparison between field-based data and results with that of HGM-RAWF. Finally, the study used nationally available spatial data to the greatest extent possible such that, in tandem with the regional adaptability of the HGM framework, the final product can serve as a proof of concept for further regional and national remote wetland functional assessment development. This paper describes the developmental framework used to create the HGM-RAWF wetland functional assessment protocol, with the resulting protocol, validation testing, and application examples provided in Backhaus (2022) and forthcoming publications.

## Materials and methods

### Existing functional assessment protocols

The HGM-RAWF development process sought to adapt methods from validated, field-based functional assessment models to the largest extent possible. Such functional assessment models were developed and calibrated in consultation with expert opinion and/or field data, providing a trusted foundation from which the remote protocol could be built, and results could be compared without the need to develop and calibrate a novel set of wetland functional models. The field-based Pennsylvania Hydrogeomorphic (HGM) Functional Assessment models from Brooks et al. (2004) and the remote sensing-based Watershed-based Preliminary Assessment of Wetland Function (W-PAWF; Tiner, 2003) were chosen as the primary basis of this work after their direct comparison in Backhaus et al. (2020). Both assessments have been designed for and/or implemented in the ecoregions of the Mid-Atlantic Region (Brooks et al., 2004; Tiner, 2005; Tiner et al., 2011) and have structure, rationales, and/or models that can be adapted for regional use nationwide.

The Pennsylvania Hydrogeomorphic Functional Assessment (Brooks, 2004; hereto called the HGM functional assessment or field-based assessment), developed by Riparia at the Pennsylvania State University, consists of conceptual models for 12 wetland functions (Table 1) adjusted for six regional HGM subclassifications (headwater floodplain, mainstem floodplain, slope, riparian depression, isolated depression, and fringing) and applicable in four Mid-Atlantic ecoregions (ridge and valley, piedmont, unglaciated plateau, and glaciated plateau). Literature of the time was used to create variables important to determining the level of each function, and these variables were, in turn, used to develop the conceptual functional models for each function and HGM subclass. Some of these variables were left as placeholders, allowing for future development of the functional assessment model and its associated sampling protocol as advances in literature and data collection permit.

**Table 1:** Comparison of functions by functional category from the wetland functional assessments used in the development of HGM-RAWF, including the Pennsylvania Hydrogeomorphic Functional Assessment (Brooks, 2004) and Watershed-based Preliminary Assessment of Wetland Function (Tiner, 2003). The functions of each system are listed in their associated column by functional category for categorical demonstration only; comparisons between functions are not intended to be made by row

Functional category	Brooks et al. (2004) functions	Tiner (2003) functions
Hydrology	F1: Energy Dissipation/Short-term Surface Water Detention	Surface water detention
	F2: Long-Term Surface Water Storage	Coastal storm surge detention
	F3: Maintain Characteristic Hydrology	Streamflow maintenance
	F4: Placeholder (for Potential Future Hydrology Function)	Shoreline stabilization
Biogeochemistry	F5: Removal of Inorganic Nitrogen	Nutrient transformation
	F6: Solute Adsorption Capacity	Retention of sediments and other particulates
	F7: Retention of Inorganic Particulates	
	F8: Export of Organic Carbon (dissolved and particulate)	
Biodiversity	F9: Maintenance of Characteristic Native Plant Community Composition	Provision of habitat for fish and other aquatic animals
	F10: Maintenance of Characteristic Detrital Biomass	Provision of waterfowl and waterbird habitat
	F11: Vertebrate Community Structure and Composition	Provision of other wildlife habitat
	F12: Maintenance of Landscape Scale Biodiversity	Conservation of biodiversity

**Fig. 1:**
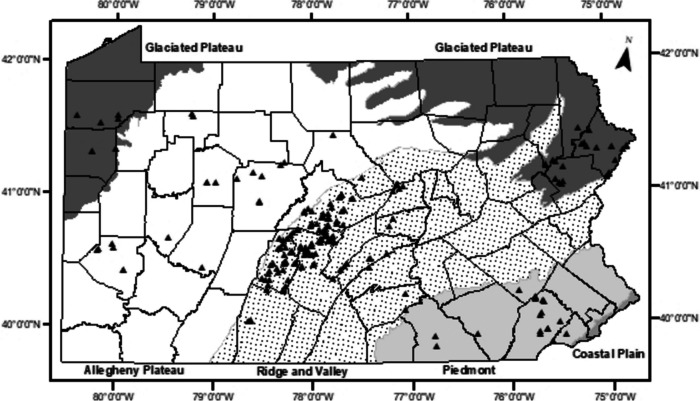
Map of the locations of Riparia’s reference wetlands in Pennsylvania, USA. The ecoregions of the state are denoted by labels and shading

An accompanying field sampling protocol was then developed to provide the best possible indicator of the variables through quantitative or qualitive measurement or observation via fieldwork or GIS (Wardrop et al., 2004). The field sampling protocol was implemented by Riparia between 1993 and 2003 as part of an intensive effort to establish a set of 222 Pennsylvania reference wetlands (Riparia Reference Wetlands Database; Fig. 1). The collection of these reference sites was designed to span a gradient of HGM subclasses, ecoregions, and human disturbance. From this, a set of reference standard sites and their accompanying reference standards were established through the identification of sites with minimal anthropogenic disturbance and high, maintained functional capacity

Each model and variable of the HGM functional assessment results in a 0–1 score by which assessed wetlands can be compared. Both the individual variable scores and the total functional score were calibrated to the reference wetlands data such that a score of 1 represents the top level of either the value of an individual variable or the functional performance one would expect of a minimally disturbed wetland of a specified HGM subclass, while a score of 0 represents a highly disturbed wetland where either the individual variable is at a level that is expected to minimally contribute to functional performance or the function is minimally performing. Differences among scores represent a conceptual, relative comparison of the value of a variable or the functional performance between wetlands. This calibration was possible given the gradient of human disturbance across ecoregions and regional HGM subclasses that are contained within the reference wetlands database. The availability of the Riparia Reference Wetlands Database also allows for remote assessment proxies to be directly compared and calibrated to ground truth data.

W-PAWF was developed as a remote functional assessment protocol that could be regionally adapted throughout the USA to assess wetlands at the watershed scale or greater. It has been implemented several times in the Mid-Atlantic Region (Backhaus et al., 2020; Tiner, 2005; Tiner et al., 2000; Tiner et al., 2011; Tiner et al., 2015) and was thus deemed appropriate for use in the development of this regional remote assessment. W-PAWF assesses 10 functions (Table 1), whose rationale for inclusion and means of assessment (i.e., variables) were qualitatively determined by expert observation and opinion. These functions are assessed using combinations of four descriptors (landscape position, landform, water flow path, and waterbody type; also referred to as LLWW) that are appended to an assessed wetland to give it HGM-like classifications (Tiner, 2014). These combinations, occasionally complemented by remote sensing interpretation of wetland and landscape characteristics, provide an absent, “moderate,” or “high” functional performance rating for each function.

Many of the functions W-PAWF assesses were directly comparable to those of the HGM functional assessment and their rationales provided potential remote sensing proxies for the HGM assessment variables in the HGM-RAWF development process. The resulting qualitative rating of W-PAWF is a less desirable result than the quantitative scores (or aggregates thereof) of the HGM functional assessment, thus relegating use of this remote assessment as a means of providing potential remote sensing proxies in this study.

### Data requirements

Field-based data was sourced from the Riparia Reference Wetlands Database. This dataset contains the field data, functional assessment variable results, and functional model results for 222 reference wetlands established throughout Pennsylvania, USA, that were sampled and assessed through the HGM functional assessment. Data was initially collected between 1993 and 2003 and spans the HGM subclasses and ecoregions of the field-based assessment.

A secondary goal of this study was to create a product that can be adapted throughout the USA for potential regional and national development, thus requiring remote proxies to be obtained from readily available public datasets to the greatest extent possible. Aerial imagery and LiDAR were widely available from state and federal sources, with timepoints spanning decades available in some areas. Federal spatial databases such as the US Fish and Wildlife Service (USFWS) National Wetlands Inventory (NWI), the Multi-Resolution Land Cover Characteristics Consortium National Land Cover Database (NLCD), and United States Department of Agriculture Natural Resources Conservation Service (USDA-NRCS) Soil Survey Geographic Database (SSURGO), available from their respective federal agencies, were used in the development of the remote assessment and their use exemplified in Table 2. Specific dataset requirements for HGM-RAWF are listed in its in Backhaus (2022).

**Table 2:** Federal spatial datasets used in the development of HGM-RAWF and examples of their potential applications in the functional assessment

Agency & dataset	Dataset description	Potential HGM-RAWF application
USFWS NWI	Spatial inventory of wetland area polygons across the USA, with characteristic descriptors such as vegetation and hydrology	Wetland/stream connectivity in biodiversity functions (development only; not in final protocol); potential source of wetland polygons for assessment
Multi-Resolution Land Cover Characteristics Consortium NLCD	30-m resolution dataset of descriptive land cover across the USA as classes (e.g., urban, agriculture, forest), percent impervious surface, and percent tree canopy	Part of several variables as proxies of floodplain obstruction (percent urban development) and coarse woody debris (percent tree canopy cover)
USDA-NRCS SSURGO	Spatial database of soil characteristics collected across the United States	Hydric soil rating, drainage classes, and soil texture as indicators of wetland biogeochemical process

### Variable development process

With the foundation of the remote functional assessment’s models established, the following workflow was developed to find and filter remote-based proxies for field-based variables and evaluate their potential for use in the HGM functional models.

#### Review of variables and functions

The proven application, quantitative results, robust assemblage of functions, and associated Riparia Reference Wetlands Database of the HGM functional assessment (Brooks, 2004) made it the preferred basis for building a remote-sensing based assessment in the region. However, as demonstrated in Backhaus et al. (2020), W-PAWF provided rationales and means by which several HGM functional models could be remotely assessed and was thus used to inform the identification of remote proxies for variables used in HGM-RAWF.

**Fig. 2:**
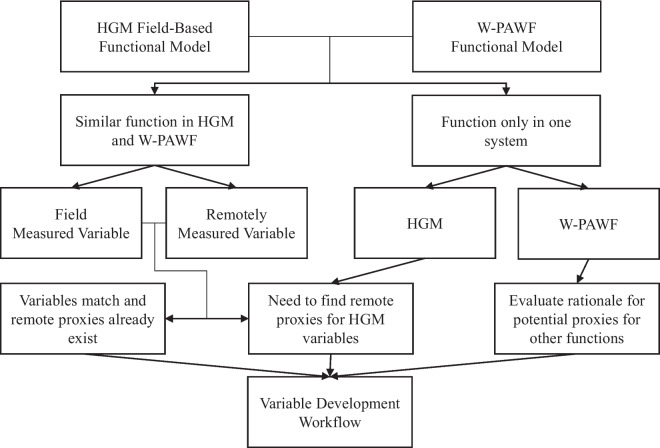
Workflow for the function and variable review process in the development of HGM-RAWF

At the functional level, the models of Brooks et al. (2004) were compared to their respective functions in the W-PAWF in a workflow illustrated in Fig. 2. As an individual functional model was evaluated, it was compared with any functional model of the W-PAWF that was determined to describe the same ecological processes and, whenever possible, similar rationales and variables used for assessment. When a function from Tiner (2003) was not directly comparable to an HGM function, its variables and rationales were individually reviewed for potential inclusion in other HGM functions.

#### Remote proxy screening and scoring

**Fig. 3:**
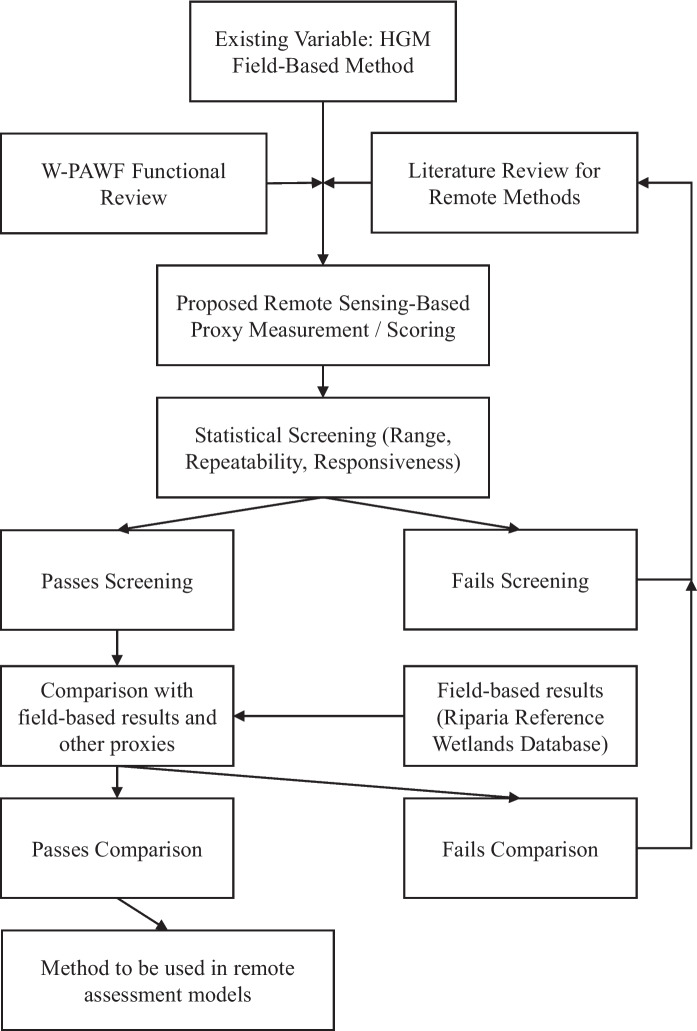
Workflow for the evaluation of variables and remote proxies for inclusion in HGM-RAWF

As the basis of the functional models, the variables of each functional model were first evaluated in a process illustrated in Fig. 3. Each variable was reviewed for the rationale of its inclusion, measurement method(s), and scoring system, reviewing from three approaches: the field-based assessment, W-PAWF, and a literature review of the variable. Using these sources, a set of potential remote proxies for the field-based variable methods were generated for each variable.

Once several potential remote proxies were established, a statistical screening process was used to evaluate the best candidate proxy for use in the remote assessment. This screening mimics that of the National Wetland Condition Assessment (NWCA) and other National Aquatic Resource Surveys of the United States Environmental Protection Agency (Stoddard et al., 2008; United States Environmental Protection Agency, 2016). The process screens candidate indicators for three criteria: *range* (skewed distributions), *repeatability* (variability within reference standard sites to all sites), and *responsiveness* (distinguish between least and most distrusted sites). Thorough descriptions of each test are available in the cited literature and Backhaus (2022), with an example of screening results for the soil redoximorphic features variable available in Table 3.

**Table 3:** Results of the screening process of potential remote proxies and the field-collected data in the development of soil redoximorphic features variable. This compared three different scored measures (soil hydric rating, drainage class, and an average of the two) to the field scores

	Hydric rating	Drainage class	Avg hydric/drain	Field score
Range	PASS-	PASS	PASS	PASS
Repeatability	S:N = 1.01	S:N = 1.01	S:N = 0.87	S:N = 0.96
Responsiveness	*p*=0.180, H=1.79	*p*=0.266, H=1.24	*p*=0.155, H=2.02	*p*=0.125, H=2.35

Each variable was screened using the results of all sites as well as groupings of the sites by HGM subclass and ecoregion. While the screening results from all sites were the most preferred for interpretation, the scoring systems of several variables were segregated by HGM subclass and/or ecoregion in the field-based assessment. Thus, the screening process was broken down to these groupings to investigate the results of the individual groups (i.e., individual HGM subclass or ecoregion) and how they differed within the screening process.

The screens were not employed to exclude any potential proxies from further revaluation but were used to guide the workflow towards a final selection of remote measurement methods. In some cases, the nature of the available data did not allow any proxy to pass all screens or multiple proxies for the same variable may have each failed a different screen (e.g., soils data typically skewed towards wetter soil conditions). In cases such as these, the original field-collected data was also screened utilizing the same tests. Where the field data could not pass the screens, it could be inferred that the remote proxies may not need to pass screens but should have similar screening results.

Once remote proxies had been determined, their measurements were translated to a 0–1 score to match the outputs of the field-based assessment. The field-based assessment’s scores were calibrated to the reference site data; as such, development of the remote proxies’ scores attempted to replicate the field scores to the greatest extent possible to take advantage of this calibration. This could involve relationships such as the direct use to field-based scoring, correlation of field data and remote sensing data through a transformation of the latter to a scoring system comparable to field score of the variable, or direct use of percentage scores plugged into the 0–1 scoring range and tested for correlation with field-based scores.

#### Selection of a final remote proxy

When multiple proxies passed the screening process for a single variable, comparisons of the screening results were made between the proxies to move towards a final selection. It would be preferred to select the screen that passed the range test and had the most desirable repeatability and responsiveness results; however, this was not always possible, as demonstrated in with the soil redoximorphic features variable in Table 3. These situations required an in-depth investigation of the proxies and their statistical relation to the original field data, relation to the scores of the variable in the field-based assessment, and/or a comparative basis from the development process of the field-based assessment itself. In general, the proxies generating similar to slightly underestimated functional scores when compared to the field-based results were preferred, as to be conservative and not overestimate functional performance. A set of standard tests and choices were not developed, given the variability in these comparisons, and instead the final selection required some level of qualitative interpretation and reasoning. Building upon the example of the soil redoximorphic features variable in Table 3, interpretation and reasoning would be as follows:

- The hydric rating results did not pass the range score, while the field data did; therefore, it was not considered viable on its own.
- Drainage class results were more repeatable than the field-based scores, but not as responsive, while an average of the hydric rating and drainage class scores were less repeatable but more responsive. Given that soils across all sites are going to score similarly to their reference standard conditions as a characteristic of their geomorphology, we would expect repeatability to be of less importance than responsiveness (where disturbance could alter long-term soil redox conditions).
- One-way analysis of variance (ANOVA) comparisons between the field scores with drainage class scores and the field scores with average hydric/drainage scores found drainage class to be significantly different than the field scores, while the average hydric/drainage scores were not.
- Given these findings, it was decided to select the average of the hydric rating and drainage class scores for their closest similarity to the field scores and their stronger responsiveness. This selection matches the rationale of the redox variable, where the extent of redox presence is a proxy of soil moisture and water storage conditions. While some correlation between the two characteristics may exist, the combination of hydric rating presence and the relative temporal presence of water due to drainage allows one to infer the relative development of redox condition formation and, when no hydric rating is present, if conditions exist for redox reactions to occur.

In cases where no remote proxies were found to be viable, their means of measurement or scoring systems were reassessed to find any potential alternatives. In the case of measurement methods, this required the workflow to return to the literature review stage to find additional remote proxies to screen and evaluate. Scoring adjustments involved changes such as statistical data transformations or adjustments of categorical scores based on the field assessment or literature. In extreme cases where no acceptable remote proxies could be found within the confines of the study (e.g., readily available data, proof of concept in wetland ecosystems and/or geographic area, etc.), standard values were developed from the field-based data. These standard values were typically average values by HGM subclass and/or ecoregion for that variable. One such example was the variable for soil organic matter, where the various remote sensing methods and spatial datasets reviewed and statistically screen and tested were unable to produce scores that adequately reflected the laboratory results of the field-based methods, and, therefore, averages of the field values by hydrogeomorphic subclass were utilized in HGM-RAWF.

As a contingency for variables where no remote sensing proxy could be directly matched with field data or results of the field-based assessment, it was directly compared with field-based function-level scores in which the variable is used. This comparison was made using a classification and regression tree (CART) regression, using the field-based results of a functional model as a response and the remote proxies’ results as predictors. The remote proxy predictor found to be of most importance by CART in most or all functions in which the variable was included was selected as the proxy to be used. One such example was the microtopography variable, where original field transects could not be located and/or replicated remotely. As the literature review for the variable determined the use of sitewide LiDAR data to be preferable over single transects, area-based measurements of surface rugosity, roughness, and/or ruggedness utilizing the LiDAR data were directly compared via CART analysis to the field-based results of functional models that incorporated microtopography, as no assessed field-based dataset of areal microtopography was available for comparison.

### Functional model assessment

As remote data was likely to be less accurate than field measurements for most variables, it was expected that each variable would contribute a degree of variability to each of the functional models’ results when compared to their respective field-based results. To ensure that the cumulative error within each functional model did not decrease the confidence in its results beyond an acceptable range, the efficacy of the remote assessment’s was tested by evaluating the differences between field-based and remote sensing-based results. A one-way analysis of variance (ANOVA) was undertaken for each function to test for significant differences between the means of the field-based functional results and the remote assessment’s results. The means of these results were further scrutinized, as significant statistical differences, or lack thereof, did not always translate to a meaningful difference in intended application (e.g., a difference in means of <0.2 on the 0–1 scoring scale may not have any ecologically significant difference for the assessment of a specific function). These comparisons were also made in tandem with a two sample Kolmogorov-Smirnov (K-S) test to detect significant differences in distribution, using the nonparametric test for its ability to describe the maximum difference in cumulative distributions. This ensured if the data had similar, meaningful means, the scores also represented the distribution of data that was set by the field data calibration of the field-based assessment.

If the means and/or distributions of the remote assessment’s results were found to be significantly different and not ecologically meaningful, the proxies were reevaluated for any potential improvements in confidence. This could be changes such an adjustment in scoring, a change in data resolution, or a return to the literature review stage to restart the variable workflow process until a more accurate set of results is achieved. Once similar means and distributions of a function’s scores were found to be acceptable, the proxies, variables, and functional models were determined to be ready for use in HGM-RAWF. Results of this workflow across all reference sites are demonstrated in Table 6, where ANOVA results for some functions resulted in a statistically significant differences; however, similar distributions and/or an ecologically meaningful difference in means were considered adequate to accept the remote proxies for the functional model.

### Sensitivity analysis

A sensitivity analysis of the functional models was provided in the development of the HGM functional assessment (Brooks et al., 2004) to examine the relative contribution of each variable to the functional models; however, the addition of a new V_WOODY_ variable (see discussion) required a re-analysis of the functional model the variable supports (F8: Export of Organic Carbon). A sensitivity analysis macro designed specifically for the analysis of HGM models by the ACOE (FCIRANGE; United States Army Corps of Engineers, n.d.), used in the HGM functional assessment development process (Brooks et al., 2004). This method changes the value of one variable while holding all others at a constant base value and generates a resulting table that provides a range for each variable for each base value, with greater range indicating a greater influence on the functional model.

The ACOE sensitivity analysis was applied to the HGM-RAWF model for F8. The results of the F8 re-analysis were combined with the analysis of the functional models for F1–F7 of the Brooks et al. (2004) analysis, given their identical functional models, to determine if any variables were strongly influencing multiple models. This influence ranking was defined as the presence of any variables that were found in multiple models with a high influence relative to the other variables.

## Results

### HGM-RAWF protocol

The aforementioned workflows were used to create a protocol by which HGM-RAWF could be used to assess wetlands classified in the HGM subclasses of the Mid-Atlantic. The final product contains 12 variables (Table 4) used in 8 functional models (Table 5). This full protocol describes the data necessary to run HGM-RAWF, outlines how this data is used to measure and score each variable, and provides the functional models in which these variables are used. Each variable description also includes details of the development process literature review, screening process, and selection process where appropriate and is available in Backhaus (2022).

**Table 4:** Variables of HGM-RAWF

HGM-RAWF variable	Description
V_BIOMASS_	Aboveground vegetative biomass
V_FLOODP_	Characteristic floodplain hydrology (placeholder)
V_GRAD_	Elevation gradient
V_HYDROCHAR_	Characteristic hydrology of groundwater supported wetlands (placeholder)
V_HYDROSTRESS_	Hydrologic stressors
V_MACRO_	Microtopographic to macrotopographic relief
V_ORGMA_	Soil organic matter (SOM) at 5cm depth
V_REDOX_	Soil redoximorphic features
V_ROUGH_	Surface roughness (Manning’s *n* roughness coefficient)
V_TEX_	Soil texture
V_UNOBSTRUCT_	Floodplain obstructions
V_WOODY_	Woody debris

**Table 5:** Functions of HGM-RAWF by functional category

Functional category	Function
Hydrology	F1: Energy Dissipation/Short-term Surface Water Detention
	F2: Long-Term Surface Water Storage
	F3: Maintain Characteristic Hydrology
	F4: Placeholder
Biogeochemistry	F5: Removal of Inorganic Nitrogen
	F6: Solute Adsorption Capacity
	F7: Retention of Inorganic Particulates
	F8: Export of Organic Carbon (dissolved and particulate)

### Remote proxy screening and functional model assessment

An example of results from the remote proxy screening process is available in Table 3. Table 6 provides results of the functional model assessment, comparing the HGM-RAWF and field-based assessment results across all reference wetlands for each functional model. Further examples of results of the remote proxy screening and functional model assessment are available in Backhaus (2022) that demonstrate the results across HGM subclasses and ecoregions.

**Table 6:** Results of the functional model assessment, statistically comparing the HGM-RAWF assessment results across all reference wetlands to their field-based counterparts in the Riparia Reference Wetlands Database through ANOVA and Kolmogorov-Smirnov tests

Function	ANOVA						K-S	
	Source of variation	SS	df	MS	F	*p*-value	K-S Test	K-S Crit
F1	Between groups	0.07	1	0.07	1.27	0.26	0.153	0.152
	Within groups	16.22	298	0.05				
	Total	16.28	299					
F2	Between groups	0.17	1	0.17	3.77	0.05	0.205	0.176
	Within groups	9.89	214	0.05				
	Total	10.07	215					
F3	Between groups	0.00	1	0.00	0.19	0.67	0.045	0.197
	Within groups	4.81	186	0.03				
	Total	4.82	187					
F5	Between groups	0.25	1	0.25	10.35	0.00	0.214	0.131
	Within groups	9.83	414	0.02				
	Total	10.07	415					
F6	Between groups	0.17	1	0.17	5.27	0.02	0.162	0.131
	Within groups	12.83	406	0.03				
	Total	13.00	407					
F7	Between groups	0.38	1	0.38	9.46	0.00	0.192	0.140
	Within groups	14.32	360	0.04				
	Total	14.70	361					
F8	Between groups	0.05	1	0.05	1.50	0.22	0.128	0.131
	Within groups	12.42	408	0.03				
	Total	12.46	409					

### Sensitivity analysis

Results of the sensitivity analysis for functional models F1–F7 can be found in Brooks et al. (2004) while the results of the F8 re-analysis are available in Appendix B of Backhaus (2022). Table 7 depicts the statistical interpretation of the sensitivity analysis for the HGM-RAWF models.

**Table 7:** Statistical interpretation of the HGM-RAWF sensitivity analysis. Values represent the count of functional models a variable was included in for a particular HGM subclass. Values in the parenthesis are the average influence ranking of the range of that variable, with 1 being the highest ranking (i.e., strongest influence on the models) and higher values indicating lower ranking, for the models the variable is included in within that HGM subclass

HGM-RAWF variable	HGM subclass		
	Headwater floodplain, mainstem floodplain	Slope	Riparian depression, isolated depression, fringing
V_BIOMASS_	1 (1.00)	1 (1.00)	1 (1.00)
V_FLOODP_	5 (1.00)	-	-
V_GRAD_	2 (2.00)	4 (1.00)	-
V_HYDROCHAR_	-	1 (1.00)	1 (1.00)
V_HYDROSTRESS_	-	1 (1.00)	3 (1.00)
V_MACRO_	4 (2.75)	3 (2.33)	-
V_ORGMA_	3 (2.33)	3 (1.67)	3 (1.67)
V_REDOX_	4 (2.50)	3 (2.00)	3 (1.67)
V_ROUGH_	3 (2.67)	3 (2.33)	1 (2.00)
V_TEX_	1 (3.00)	1 (2.00)	1 (2.00)
V_UNOBSTRUCT_	5 (2.40)	-	-
V_WOODY_	1 (3.00)	1 (2.00)	1 (2.00)

## Discussion

The framework described in this paper provides a foundation for the development of an HGM-RAWF protocol through its hydrogeomorphic approach, workflows, quantitative methods, and qualitative reasoning that can be adapted regionally with only minor changes for regional considerations and accommodation. However, each regional iteration will need to consider the implications of its selected field-based data, field-based and remote assessment methods and protocols, desired goals and scoring methods, etc. on the development process and application of its protocol. The following discussion focuses on the assumptions, considerations, and limitations that arose from the development of the Mid-Atlantic Region HGM-RAWF and their effect on the final protocol and its use. These are provided as an example of what may arise when developing a new protocol through the framework, but will vary for each individual application of this development process.

### Contrasts of Brooks et al. (2004) and HGM-RAWF

As the workflows for functions and variables were conducted, it became apparent that HGM-RAWF would be unable to assess certain variables and functions, even with the input from W-PAWF and literature review. Biodiversity functions and their derivative variables proved to be particularly problematic in terms of their data specificity. For example, F9: Maintenance of Characteristic Plant Community Composition is comprised of a vegetative species-specific adjusted floristic quality index (FQAI), a cover of exotic species, and a measurement of forest regeneration. Even in the field, identification of high-quality species that drive the FQAI score can be difficult, and fine resolution data would be needed with frequent revisit time to capture invasive species during the growing season and the presence of regeneration in the understory during leaf-off (with further difficulty of differentiation small snags and woody debris from seedlings and saplings). In addition, while FQAI does correlate with the disturbance score of the Riparia reference sites (Brooks, 2004), this relationship was not strong enough to be generalized and still provides confidence at site-specific level as a remote proxy. As such, HGM-RAWF does not include the biodiversity functions included in the field-based assessment (F9–F12).

It is recommended that those requiring assessment of biodiversity functions utilize function models independent of HGM-RAWF and its associated data. Efforts such as the Gap Analysis Project (e.g., United States Geological Survey, n.d.) provide species-specific habitat models while ecosystem services models such as InVEST (Natural Capital Project, 2022) can indirectly assess some biodiversity functions through the ecosystem services the function supports. Landscape ecology concepts may also provide measures of biodiversity interest at broader scales.

Another difference between the field-based and remote assessments was the creation of the V_WOODY_ variable and the adjustments to its associated functional models. Much like the biodiversity variables, fine woody debris, coarse woody debris, and snags were difficult to remote sense. However, it was noted that these variables were always grouped within the functional models, none being independently used in other functions. These variables were included in the form

1 $$\dots {V}_{FWD}+\left(\left({V}_{CWD- BA}+{V}_{CWD- SZ\Big)}\right)/2\right)+{V}_{SNAGS}\dots$$

with these variables averaged with over variables in their parenthetical section of the equation. As such, V_WOODY_ was created by averaging Eq. 1 and substituting it into the functional models, with field results processed in a similar manner for comparison. The functional models were also adjusted accordingly as to result in the same overall functional model. With this change, we were able to correlate V_WOODY_ directly to NLCD tree canopy cover, and it was able to be included in the final protocol without any changes to the variables or functions that would result in a recalibration of their results.

### Sensitivity analysis

The sensitivity analysis provided insight into the driving variables of the functional models for each subclass through their more frequent presence in, and high influence on, the functional models. Headwater floodplains and mainstem floodplains are influenced primarily by V_FLOOPD_ (in 5 of 6 functions), slopes by V_GRAD_ (4 out of 6 functions), and riparian depressions, isolated depressions, and fringing wetlands by V_HYDROSTRESS_ (3 of 4 functions). Of note, F5 was influenced directly in all functions by the same three variables, separate from those listed above, making the aforementioned variables the dominant variables of every function other than F5 present in those subclasses (except F3 for slopes).

The relative influence has implications in HGM-RAWF due to the scoring system of these variables. V_FLOODP_ is currently a placeholder and is scored at 1.0, causing a potential overestimation of floodplain function through its relative influence on the floodplain models and given high score. While the other two HGM subclasses are not as dominated by a single variable, half of F3’s score is influenced by V_HYDROCHAR_, the other placeholder variable, and it may be overestimating the characteristic hydrology of groundwater reliant wetlands. Additionally, V_ORGMA_, a standardized, average score of results from the field-based assessment when used in HGM-RAWF, is ranked high in nearly all biogeochemistry functions in all subclasses, driving scores more towards its mean. While the HGM-RAWF results from the development process are considered acceptable in comparison to the field-based assessment results, interpretation of the results of both assessments should consider these influences.

The sensitivity analysis also revealed potential overparameterization of two of the biogeochemistry models. The hydrology models tended to have a balanced influence of variables, with V_FLOODP_ acting as a more dominant driver when it was included, and this relationship was also seen in F5 and F7. In contrast, the models of F6 and F8 (in both HGM-RAWF and HGM functional assessment) tended to include several variables with low influence. For example, the range of V_ROUGH_, V_REDOX_, and V_MACRO_ were more than 5 times smaller than V_GRAD_ for slope wetlands in F6, with these three variables providing little influence on the overall model. Further investigation should be undertaken to see if the models can be improved, potentially removing or consolidating variables and testing for any significant change in response (e.g., V_ROUGH_ and V_MACRO_ may encompass similar processes).

### Functional model limitations

#### Assumptions

Users of HGM-RAWF should consider the assumptions upon which it is built. The field-based assessment development process was started through the identification of functions deemed to be of importance by its authors. Working from these functions, the required scientific data to assess these functions was surmised and used to develop the conceptual functional models. It was from these models and required data that the field sampling protocol was developed, and the results of this protocol were used to calibrate the scoring of the functional models. Each of these steps incorporated some level of assumption, be it which data was indicative of functional performance, the relative weights of variables in the functional models, the ability of the field sampling data to provide a trusted scoring calibration, etc.

HGM-RAWF compounds these assumptions by adopting the framework of the field-based assessment and building upon them through the development of remote proxies that are calibrated to the field-based assessment’s results. While the HGM-RAWF development process undertook a modern literature review to source remote proxies and compared them to the field-based results, the underlying rationales of the field-based assessment are still based in its own developmental literature review in the 1990s. Potential remote proxies that are more representative of a real-world functional performance may have been filtered out in favor of one closely mirroring the decades-old field data and methods.

Future research should seek to revisit the development process of the HGM functional assessment. This could involve changes such as adding or removing functions, adjusting the weights of variables in the functional models, updating the field sampling protocol, and/or the collection of new data based on methods from a current literature review. It may be found that the current variables are no longer the strongest predictors of a function and its performance or that a different combination of variables and their weights provide a stronger conceptual model for a function. For example, the HGM-RAWF literature review found water presence in soil pore spaces to be a driver of nitrogen transformation (F5). While V_REDOX_ was a strong proxy for the presence of these reactions in the development of the field-based assessment, soil moisture measurements in the field or from a remote sensing platform may prove to be stronger representation of the biogeochemical reactions of the function in an updated functional model.

Despite its assumptive limitations, the continued application and adaptation of the HGM functional assessment in academic and regulatory applications (e.g., Backhaus et al., 2020; Hychka et al., 2007; Pennsylvania Department of Environmental Protection, 2016; Pennsylvania Department of Environmental Protection, 2022; Wardrop, Kentula, Jensen, et al., 2007a; Wardrop, Kentula, Stevens, et al., 2007b) demonstrates it is a trusted methodology. With nearly similar results to the field-based assessment, that trust should extend into the use of HGM-RAWF until further development efforts are undertaken to provide alternative, updated methodologies.

#### Data constraints

The goals of the development process put several constraints on the final protocol, namely from the restriction to readily available public data. While this data was adequate for the rationales of the existing GIS-based functional assessment methods, many of the remote methods identified during the literature review stage were dependent on finer resolution data. This data was typically required for not only detection of the desired variable, but was often itself used in specialized processing necessary to detect the variable. For example, high-resolution LiDAR point clouds were not readily interpretable and needed intensive processing to filter the data to detect desired objects such as coarse woody debris. However, these methods were constrained by computing requirements, required ground truth data, and were untested in wetland ecosystems and/or over broad geographic areas. Further research should build these methods towards more general use with readily available data and additional ground truthing, such that variables can be more accurately used in the remote assessment.

Use of widely available data also constrained the temporal available of data for the development of HGM-RAWF. Data such as fine-resolution imagery (i.e., < 1 m pixels) and LiDAR are only collected once every several years by public sources, and spatial databases such as the NLCD are updated at 5-year intervals. This can leave a disconnect not only between the time of the assessment and when the remote data was collected, but also between the datasets being utilized in the remote assessment. One must be careful where land use or landcover may differ from present conditions or that of utilized datasets when interpreting assessment results.

In addition to the remote data limitations, the Riparia Reference Wetlands Database contained several statistical gaps during the development of Brooks et al. (2004) that carried into the development of the remote assessment. These gaps are not believed to have a detrimental effect on the overall results but should be considered during interpretation of HGM-RAWF’s results (Backhaus, 2022). Future development of the assessment should consider additional collection of field data to address these statistical gaps.

## Conclusions

With its development based in the HGM framework of the Brooks et al. (2004) and a statistical foundation in the data of the Riparia Reference Wetlands Database, the Mid-Atlantic Region iteration of HGM-RAWF is expected to provide specific, quantitative functional scores at scales where field-based methods are time and cost prohibitive and where existing remote-based assessments are constrained to limited categorical scoring. Through the HGM-RAWF developmental framework, the resulting remote-based protocol’s results are expected to be like those of its field-based counterpart; therefore, we deem HGM-RAWF to be an acceptable desktop functional assessment for use in the geographic region in which it is designed for. Though some limitations and data gaps exist, similar shortcomings are present in the field-based assessment, while it has been successfully implemented in its intended ecoregions, and thus, HGM-RAWF is considered adequate for use. A study of the remote assessment’s efficacy at varying scales (e.g., site-specific, watershed, regional, etc.) and functional levels (i.e., individual functions, functional category, and site-level functional assemblage) is forthcoming, and new reference sites have been collected to begin to address data limitations and serve as testing locations for the protocol.

The use of nationally available spatial data within HGM-RAWF also addressed the secondary goal of the development process. With the regional adaptability of the HGM framework, achieving this goal allows HGM-RAWF to serve as a regional, conceptual proof of concept that outlines further development of remote wetland functional assessment protocols for use nationwide. Such a national functional assessment process, in tandem with national assessments of wetland area and condition, provides potential means by which changes in the nation’s wetlands, and their associated ecosystem services could be observed and holistically understood.

## Data Availability

Data from the Riparia Reference Wetlands Database is available upon request from their website (https://riparia.psu.edu). Data resulting from the HGM-RAWF development process is intended to be archived in the Penn State Data Commons.
